# The prevalence of depressive symptoms among fathers and associated risk factors during the first seven years of their child’s life: findings from the Millennium Cohort Study

**DOI:** 10.1186/s12889-016-3168-9

**Published:** 2016-06-13

**Authors:** Selina Nath, Lamprini Psychogiou, Willem Kuyken, Tamsin Ford, Elizabeth Ryan, Ginny Russell

**Affiliations:** Institute of Psychiatry, Psychology & Neuroscience (IoPPN), King’s College London, De Crespigny Park, Denmark Hill, London, SE5 8AF UK; Mood Disorders Centre (MDC), Psychology, College of Life and Environmental Sciences, University of Exeter, Exeter, EX4 4QG UK; Department of Psychiatry, University of Oxford, Warneford Hospital, Oxford, OX3 7JX UK; Institute of Health Research, South Cloisters (St Luke’s Campus), University of Exeter Medical School, Exeter, EX1 2 LU UK

**Keywords:** Paternal, Fathers, Depressive symptoms, Unemployment

## Abstract

**Background:**

Increasing evidence suggests that postnatal paternal depression is associated with adverse emotional, behavioural and cognitive outcomes in children. Despite this, few studies have determined the prevalence of fathers’ depressive symptoms during the first few years of their children’s lives and explored what factors are related to these symptoms. We estimated the prevalence and examined associated risk factors of paternal depressive symptoms in a nationally representative sample of fathers with children aged between 9 months and 7 years old from the Millennium cohort study. The risk factors examined were maternal depressive symptoms, marital conflict, child temperament, child gender, paternal education, fathers’ ethnic background, fathers’ employment status, family housing, family income and paternal age.

**Methods:**

Secondary data analysis was conducted using the UK Millennium cohort study, which consisted of data from England, Scotland, Wales and Northern Ireland of families with infants born in the year 2000/2001. Data from four sweeps were used from when children in the cohort were aged 9 months, 3, 5 and 7 years old (*n* = 5155–12,396).

**Results:**

The prevalence of paternal depressive symptoms over time was 3.6 % at 9 months, 1.2 % at 3 years old, 1.8 % at 5 years and 2.0 % at 7 years (using Kessler cut-off points to categorise high depressive symptoms vs low depressive symptoms). Linear regression trends (using continuous measures of depressive symptoms) indicated that both paternal and maternal depressive symptoms decreased over time, suggesting similar patterns of parents’ depressive symptoms after the birth of a child, but the decrease was more evident for mothers. Paternal depressive symptoms were consistently associated with fathers’ unemployment, maternal depressive symptoms and marital conflict. Socioeconomic factors such as rented housing when child was 9 months and low family income when child was 5 and 7 years were also associated with higher paternal depressive symptoms.

**Conclusions:**

Paternal depressive symptoms decreased among fathers when their children were aged between 9 months to 3 years old. Paternal unemployment, high maternal depressive symptoms and high marital conflict were important risk factors for paternal depressive symptoms. In light of our findings, we would recommend a more family centred approach to interventions for depression in the postnatal period.

## Background

Despite government policy and research acknowledging the importance of fathers in children’s development, parenting programs and interventions are still primarily targeted at mothers [1–5]. Growing evidence suggests that the postnatal period may be associated with higher prevalence of depressive symptoms in fathers as well as in mothers and may also be associated with adverse emotional, behavioural and cognitive outcomes in children [6–11]. To understand the influence of paternal depression on children’s emotional, behavioural and cognitive outcomes in more depth, we need to determine fathers’ depressive symptoms during the first few years of their children’s lives and the associated risk factors. This might enable clinicians to identify when effective interventions can be implemented for the best developmental outcomes in both children and adults, as well as indicating to what extent the development of paternal depressive symptoms itself may be related to sensitive periods after birth. The aim of the current paper is to describe the fluctuation in paternal depressive symptoms within the first 7 years of their children’s lives and the associated risk factors using a nationally representative sample of fathers.

Research on paternal depressive symptoms has mostly focused on the prevalence of symptoms during the first year after a child’s birth as this is a sensitive period in which parental depressive symptoms may influence children’s development [8, 12]. The prevalence of paternal depressive symptoms from the first trimester to 12-months after birth has been reported as 10.4 % from a meta-analysis consisting of 43 studies, where the highest rates of depressive symptoms were reported amongst fathers when their infants were 3–6 months old [9]. However, an integrative review of 20 studies reported a more varied prevalence ranging between 1.2–25.5 % during the postpartum period [12]. These variations in the rates of paternal depressive symptoms are likely to be due to the use of different assessment methods and populations in previous research. Studies that conduct clinical interviews might under-represent paternal depression compared to self-report measures and some studies have utilised liberal cut-off points which may over represent mild depressive symptoms in the clinical range [9, 12]. In addition, these studies also used a sample of fathers that were predominantly Caucasian, so the finding may not be applicable to the general population consisting of ethnic minorities.

Knowledge about paternal depressive symptoms past the first year of child’s life is limited. In the Longitudinal Study of Australian Children (LSAC) 1.9 % fathers reported psychological distress measured using the Kessler Scale when their child was 3–12 months old, 1.4 % when their child was 2–3 years old and 2.2 % when their child was 4–5 years old [13]. This study showed relatively little change in paternal depressive symptoms rates over time. The authors acknowledged that this might be an underestimation of paternal depressive symptoms as the sample was not representative of socioeconomically disadvantaged fathers in Australia. Using a more representative sample of 7247 fathers participating in the Medical Expenditure Panel Survey (MEPS) from the USA, paternal depressive symptoms using the Patient Health Questionniare-2 (PHQ-2) were reported at 6.19 % in fathers with children aged 5–17 years [14]. Another study from the USA, using the National Longitudinal Study of Adolescent Health, found that resident fathers’ depressive symptoms increased from child birth until 5 years of age, followed by a decrease between 5 and 10 years of age [15]. The current study will build on the literature by investigating paternal depressive symptoms from 9-months to 7 years old, using a large nationally representative sample of fathers in the UK including ethnic minorities, which should lead to more consistent findings compared to the mixed reports in previous literature.

To gain a better understanding of paternal depressive symptoms and prevention methods, we need to determine associated risk factors. Many studies have reported high correlations between maternal and paternal depressive symptoms with up to 50 % of fathers likely to experience depressive symptoms if their partner is experiencing depression [9, 12, 16–18]. This could be due to the direct influence of mothers’ mental states on their partners, that both parents are exposed to other similar socioeconomic factors that predispose them to become depressed or depressive symptoms being correlated from the beginning of their relationship due to assortative mating patterns [19]. Therefore, it is important to compare the levels of paternal and maternal depressive symptoms over time, as it is important to target both parents in interventions. Marital conflict has also been identified as a significant risk factor for paternal depression [20, 21] and Giallo et al. [16] reported higher psychological distress amongst fathers in unhappy marital relationships.

Child factors may also influence paternal depressive symptoms including child’s temperament and gender [22]. Using data from the Avon Longitudinal study of Parents and Children, Hanington and Colleagues [23] reported paternal depressive symptoms during the first year of an infant’s life to be associated with more difficult child temperament when boys were 2 years old. There was no significant relationship for daughters. Other research has also shown paternal depressive symptoms to be associated with behavioural outcomes in boys but not for girls [7]. This might be because fathers spend more time with their sons compared to their daughters as they may identify more with them and feel that they are able to participate in more joint play activities [10, 24].

Socioeconomic factors have also been associated with paternal depressive symptoms such as unemployment, poverty, younger age, low educational level, low income and ethnicity [14, 25–27]. The UK recession that commenced in 2008-09 impacted many families, and the resultant financial and social hardship may potentially have influenced the development of paternal depressive symptoms [28, 29]. It is, therefore, important and timely to investigate the association of paternal depressive symptoms with socioeconomic variables, such as unemployment, household income, and housing.

Our primary objective is to estimate the prevalence of depressive symptoms in the UK population of fathers using data from the Millennium cohort study when children were 9 months, 3, 5 and 7 years old. In addition, we assess the association between paternal depression, family and socioeconomic contextual factors. We hypothesise that paternal depressive symptoms would be higher immediately after birth and decrease over time and that paternal depressive symptoms would be strongly associated with maternal depressive symptoms, higher marital conflict and difficult child temperament. We also predict that paternal depressive symptoms will be associated with low socio-economic factors.

## Methods

### Sample

Secondary data analysis was carried out using the first four waves of the Millennium Cohort Study (MCS). The MCS is large-scale survey of infants (*n* = 19,519) born in four constituent countries of the United Kingdom [30]. Full details of the survey, objectives, content of survey and sampling can be found in the documentation attached to the data deposited with the UK Data Archive at Essex University (UK Data Archive 2004 and 2006). The sample design allowed for over-representation of families living in areas with high rates of child poverty or high proportions of ethnic minorities in England and the three smaller countries (Northern Ireland, Wales, and Scotland). More details on the sampling strategy can be found elsewhere [31, 32].

The first wave (MCS1) of data was collected from 2001-2002 on 18,533 families, with a total of 18,819 infants aged between 9–11 months. The same sample were then invited to follow-up (MCS2) when the children were approximately 3 years old. 14,898 families from MCS1were followed-up and 692 new families were recruited into the cohort at MCS2. This totalled to 15,590 families in the second wave (MCS2) when the children were approximately 3 years old. 15,246 families were then followed up in the third wave (MCS3) when children were approximately 5 years old and 13,857 in the fourth wave (MCS4) when children were approximately 7 years old. The reduction in overall sample was due to drop-out. According to the MCS technical report of responses, families from socio-economically disadvantaged or ethnic backgrounds had higher attrition rates compared to socio-economically advantaged families [32].

Biological mothers were identified from main respondents interviews and biological fathers from partner respondents. Step-fathers and part-time resident fathers were excluded from the current study as there was not a large enough sample size for analysis of these groups. Fathers who were main respondents were also excluded to simplify analysis since main and partner questions were not always the same. Given the small numbers of twins or triplets in the sample, multiple births were excluded so that only one child per family was studied to avoid the need to include an extra level of analysis that accounted for intra-family variability. Table 1 shows the sample size of biological mothers and fathers who took part across all sweeps and used for analysis.

**Table 1:** Sample size of biological mothers and fathers across all sweeps in the Millennium Cohort Study

Parent	Sweep 1 (9 months)	Sweep 2 (3 years)	Sweep 3 (5 years)	Sweep 4 (7 years)
	N	N	N	N
Mothers	18,497	14,645	12,792	12,175
Fathers	12,882	11,253	9710	8803

### Measures

#### Paternal depressive symptoms

Rutter’s 9-item Malaise Inventory (sweep 1) was used as an indicator for depressive symptoms [33]. This is the shortened version of the Rutter’s 24-item Malaise Inventory self-completion questionnaire [34] measuring psychological distress. The 9 items selected were based on items with the highest loading first principle factor and showed adequate reliability of at least 0.70 using Cronbach’s alpha coefficient[35].

The Kessler 6 (K6) scale (sweeps 2–4) [36] was used as an indicator of current depressive symptoms in sweep 2, 3 and 4. It has an internal consistency and reliability of 0.89 using the Cronbach’s alpha coefficient [36, 37] and has shown to detect current depression [38]. We used a strict cut-off of ≥13 commonly taken to indicate clinical levels of distress [13]. Continuous scales on depression scores were created. Cut-off points were then used to determine categorical groups of K6 scores 0–12 (low depressive symptoms) and 13–24 (high depressive symptoms).

#### Family context factors

Maternal depressive symptoms (Sweeps 1–4) were measured using the Rutter Malaise and Kessler scales as above. Groups of mothers with high versus low levels of depressive symptoms were derived by applying the same method that was used for fathers.

Marital conflict was measured at all sweeps using the modified version of the Golombok Rust Inventory of Marital State [35, 39]. The original 28-item questionnaire had high content validity and reliability of 0.91 (men) and 0.87 (women) using the Cronbach’s alpha coefficient. The MCS selected seven items at sweeps 1, 2 and 3, and three items at sweep 4. Higher scores indicated higher levels of marital conflict. The scale was standardised (Mean = 0, SD = 1) and mean scores for each sweep were derived for analysis.

Children’s temperament was measured at Sweep 1 using mothers’ reports on the Carey Infant temperament scale [40]. Fourteen questions from the original scale were used, which included items on subscales of infant’s mood (five items), regularity (four items), and adaptability (5 items). On a 5-point scale (almost never, rarely, usually does not, often, almost always), higher scores indicated easier infant temperament and lower scores indicated more difficult temperament. All scores were on a continuous scale ranging from (14–70) consisting of the total score of all three subscale categories including mood, adaptability and regularity.

#### Socioeconomic factors

Paternal education and fathers’ ethnic background were reported at sweep 1. Fathers chose one of the following options: Higher degree, First degree, Diplomas in higher education, A / AS / S levels, O level / GCSE grades A-C, GCSE grades D-G, Other academic qualifications (incl. overseas) or None of these qualifications. Answers were reduced to two categories of no qualification or school level, degree or higher.

Fathers’ response on ethnic background was captured in four categories: White, Indian, Pakistani & Bangladeshi, Afro-Caribbean & British afro-Caribbean and Mixed, and Other Ethnicity.

Housing, family income, poverty indicator, fathers’ employment and paternal age were all reported at sweeps 1–4. Family housing was categorised into two categories; rented and home owner. Both mothers and fathers were asked about the household income. Equivalised family income was derived at each sweep (adjusted for the number of children per family), with household classed as living in poverty if their income was equal to or less than 60 % of the median household income for the UK. The definition of poverty set by the UK government [41]. Fathers indicated if they were currently doing paid work, had a paid job but on leave, had worked in the past but no current paid job or never had a paid job. This information was used to create a derived variable stating if respondent was in employment at each sweep. Paternal age was reported by fathers in the partner interview questions at each time point of the study. There were no fathers in the 12–19 years age category after sweep 1.

### Statistical analysis

As the sample was stratified, sampling weights were used in all analyses. This adjusted for the disproportionate number of ethnic minorities and low socio-economic participants initially recruited into the sample at sweep 1, therefore making the sample representative of the UK population. It also accounts for the effect of attrition by sweep 4. All analyses were conducted using Stata for Windows version 13 [42].

#### Fathers’ depressive symptoms across the four waves

Trends of depression during the early years of childhood were assessed using scores from the Kessler and Rutter Malaise. To make these scores comparable, Rutter Malaise scores were recalibrated from a 9-time scale to a 24-time scale to fit the Kessler scale, creating a continuous scale of depressive symptoms at each time point. The continuous scales at each time point were split into dichotomous scales of high and low depressive symptoms using the Kessler clinical cut-offs to determine prevalence of depressive symptoms among mothers and fathers over time. Data from biological parents who were full-time resident in household and reported depressive symptoms at all four time points (complete data) were used for analysis. Therefore, there were 9611 biological mothers full-time resident in the house, and 5220 biological fathers who were full-time resident in household who reported depressive symptoms scores at all time points.

A linear trend analysis was conducted for mothers and fathers to compare the trend of depressive symptoms in the population as a whole by treating depression as a continuous variable and analysing over time.

As a sensitivity analysis to explore whether the linear trend in depressive symptoms was robust to the effect of birth of further children after the cohort member, the linear trend analysis was repeated on a sample that was limited to parents with no subsequent births. This sample comprised 5612 full cases for mothers’ depression scores and 2845 full cases for fathers’ depression scores.

#### Family context and socioeconomic factors associated with paternal depressive symptoms

The influence of family context and socio-economic factors used all cases available for the following analysis ranging from 5155 to 12,396 cases depending on the variable used. Mean scores and proportions of each predictor for low depressive symptoms (K6 scores 0–12) and high depressive symptoms (K6 scores 13–24) were plotted for each time point. Then, to check whether predictors were associated with paternal depressive symptoms, we conducted a series of linear regressions using the continuous scale of paternal depressive symptoms scores as outcome, and family context and socio-economic factors as predictors in an unadjusted univariate analysis. Predictors that were significantly associated with paternal depressive symptoms (*p* < 0.01) were taken forward into multivariable regression to test which covariates were independently associated with paternal depression. Finally, predictors that were significantly associated with paternal depressive symptoms in the multivariable regressions were taken forward into one mixed effects model in Stata (using xtmixed command).

## Results

### Fathers’ depressive symptoms across the four waves

**Fig. 1:**
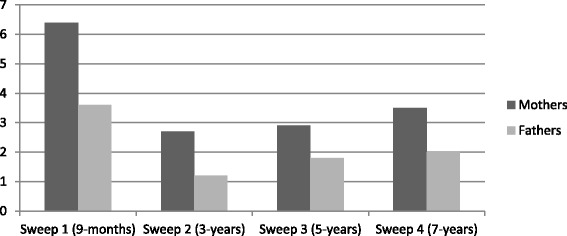
Percentage of mothers (*n* = 9611) and fathers (*n* = 5220) with high levels of depressive symptoms for biological full-time resident mothers and fathers across time in the Millennium Cohort Study (full-cases and weighted)

The prevalence of paternal depressive symptoms over time as defined by the Kessler cut-off point was 3.6 % at 9 months, 1.2 % at 3 years, 1.8 % at 5 years and 2.0 % 7 years (Fig. 1).

Mothers’ (4.09, 3.10, 2.95, and 3.01) and fathers’ (3.27, 2.71, 2.77, and 2.89) mean scores for each sweep were used to construct a linear trend. The linear trend showed a significant decrease in depressive symptoms over time for mothers (beta = -0.19, CI: -0.20 – -0.17, *p* **<** 0.001) and fathers (beta = -0.06, CI: -0.08 - -0.04, *p* **<** 0.001), but the decrease was greater for mothers than for fathers. Among mothers, depressive symptoms declined from 9 months to 7 years old, whereas among fathers, the difference was less marked although the decrease was statistically significant according to a linear model.

The sensitivity analysis excluded children who had younger siblings (42 %) to check whether the observed patterns were affected by subsequent births. The linear trends remained for both mothers (beta = -0.21, CI: -0.23 - -0.18, *p* **<** 0.001) and fathers (beta = -0.08, CI: -0.01 - -0.05, *p* **<** 0.001) with depressive symptoms significantly decreasing over time and slightly stronger than those with subsequent children. Therefore, the exclusion of subsequent births seemed to strengthen the linear trend, as subsequent births may have contributed to the maintenance of depressive symptoms over time.

### Family context and socioeconomic factors associated with paternal depressive symptoms

#### Descriptive statistics

Table 2 shows differences between fathers with low and high levels of depressive symptoms according to each predictor. Across all time points, fathers with high levels of depressive symptoms had partners with higher mean depression scores, higher levels of marital conflict, children with a more difficult temperament and were more socio-economically disadvantaged compared to fathers with lower levels of depressive symptoms.

**Table 2:** Descriptive statistics of fathers with low and high levels of depressive symptoms

	Sweep 1(9 months old) ^c^		Sweep 2 (3 years old) ^d^		Sweep 3 (5 years old) ^e^		Sweep 4 (7 years old) ^f^	
	depressive symptoms		depressive symptoms		depressive symptoms		depressive symptoms	
Predictors ^a^	Low	High	Low	High	Low	High	Low	High
Family context factors								
Maternal depressive symptoms: Mean(SD)	3.96 (4.3)	6.18 (5.6)	2.82 (3.2)	4.98 (4.6)	2.62 (3.2)	4.23 (4.6)	2.57 (3.2)	4.69 (4.4)^b^
Marital conflict: Mean (SD)	−0.04 (1.0)	0.97 (1.3)	−0.00 (1.0)	1.38 (1.3)	−0.01 (1.0)	1.13 (1.5)	−0.02 (1.0)	1.02 (1.5)^b^
Child temperament: Mean(SD)	57.44 (6.1)	55.26 (6.8)	57.49 (6.0)	56.88 (7.1)^b^	57.52 (6.0)	56.44 (6.8)	57.41 (6.0)	55.45 (6.7)^b^
Child gender (%)								
1. Boy	52	50	50	51	51	57	51	51
2. Girl	48	50	50	49	49	43	49	49
Socio-demographic factors								
Paternal education (%)								
1. No qualifications	14	31	12	29	12	37	12	27
2. School level, Degree or higher	86	69	88	71	88	63	88	73
Fathers ethnic background (%)								
1. White	91	88	92	88	93	89	92	86
2. Indian, Pakistani & Bangladeshi	5	7	5	7	4	7	4	9
3. Afro-Caribbean & British afro-Caribbean	2	2	2	3	1	0	2	1
4. Mixed, other ethic	2	3	2	2	2	4	2	4
Fathers’ employment (%)								
1. Employed	92	74	94	74	95	63	95	61
2. Unemployed	8	26	6	26	5	37	5	39
Housing (%)								
1. Council or private rent	22	43	17	40	15	46	16	46
2. Home owner	78	57	83	60	85	54	84	54
Family income: Mean (£ per week)	370.15 (205.00)	290.92 (197.10)	413.88 (227.14)	291.21 (192.93)^b^	439.95 (222.63)	272.53 (150.63)	474.08 (229.37)	309.37 (200.71)^b^
Paternal age (years): Mean (SD)	33.2 (6.0)	32.7 (6.6)	35.7 (5.9)	34.7 (6.5)	37.8 (5.9)	37.6 (6.2)	39.7 (5.9)	38.7 (6.3)^b^

^a^ Sample size ranged from 5491 to 12,396 due to missing data in the variables

^b^ SD were taken from data out of survey, as survey set in stata was giving inaccurate result of 0 SD’s

^c^ Sample size was 12,396 (high depressive symptoms = 1133, low depressive symptoms = 11,263)

^d^ Sample size was 8768 (high depressive symptoms = 129, low depressive symptoms = 8768)

^e^ Sample size was 8312 (high depressive symptoms = 165, low depressive symptoms = 8147)

^f^ Sample size was 7382 (high depressive symptoms = 179, low depressive symptoms = 7203)

#### Unadjusted univariate analysis

In the unadjusted analysis, higher levels of paternal depressive symptoms were significantly associated with higher levels of maternal depressive symptoms, higher levels of marital conflict, more difficult child temperament, unemployment, living in rented accommodation and lower family income (*p* < 0.01, Table 3) at all sweeps. Paternal age was significantly associated with depressive symptoms at sweep 1 only, with higher depressive symptoms among younger fathers. Paternal education was significantly associated with paternal depressive symptoms at sweep 1 and 3, with more depressive symptoms amongst those with no qualifications. Compared to white fathers, Indian Asian and mixed fathers reported more depressive symptoms, whereas Afro-Caribbean fathers reported fewer depressive symptoms.

**Table 3:** The associations between predictors and paternal depressive symptoms in the Millennium Cohort Study

Predictors at each sweep	Unadjusted^a^		Adjusted^b^	
	Coefficient (95 % CI)	*p*	Coefficient (95 % CI)	*p*
Predictors (Sweep 1 when child 9-months old)				
*Family context factors*				
Maternal depressive symptoms	0.17 (0.15-0.19)	<0.001	0.11 (0.08 - 0.13)	<0.001
Marital conflict	1.18 (1.18-1.38)	<0.001	1.12 (1.01 – 1.23)	<0.001
Child temperament	-0.05 ( -0.07- 0.03)	<0.001	-0.01 (-0.03- -0.00)	0.097
Child gender	-0.01 (0.13 – 0.04)	0.133		
*Socioeconomic factors*				
Paternal education		<0.001		0.011
No qualifications	reference group			
School level, Degree or higher	-0.17 (-1.42- -0.92)		-0.45 (-0.79- -0.10)	
Ethnic background		0.028		
White	reference group			
Indian, Pakistani, & Bangladeshi	0.23 ( -0.10- 0.56)			
Afro-Caribbean & British afro-Caribbean	-0.80 (-1.46- -0.14)			
Mixed, other	0.14 ( -0.42- 0.71)			
Fathers work status				
Employed	reference group			
Unemployed	1.94 (1.56-2.32)	<0.001	0.94 (0.41 -1.46)	<0.001
Housing tenure		<0.001		<0.004
Council and private renting, %	Reference group			
home owner, %	-1.40 (-1.62- -1.18)		-0.41 (-0.69- -0.13)	
Family income (per £1000 p.a.)	-2.53 (-2.98- -2.08)	<0.001	-0.35 (-0.90-0.21)	0.219
Paternal age	-0.40 (-0.55- -0.25)	<0.001	-0.08 (-0.25-0.10)	0.395
Predictors (Sweep 2 when child 3 years old)				
*Family context factors*				
Maternal depressive symptoms	0.17 (0.14-0.20)	<0.001	0.09 (0.05 -0.12)	<0.001
Marital conflict	1.14 (1.05-1.23)	<0.001	1.06 (0.95-1.17)	<0.001
Child temperament	-0.03 (-0.04- -0.01)	<0.001	-0.01 (-0.02-0.01)	0.321
Child gender	-0.08 (-0.21- 0.06)	0.273		
*Socioeconomic factors*				
Paternal education		0.013		
No qualifications	reference group			
School level, Degree or higher	-0.30 (-0.53- -0.06)			
Ethnic background		<0.001		0.017
White	reference group			
Indian, Pakistani, & Bangladeshi	0.80 (0.45-1.15)		0.60 (0.03-1.18)	
Afro-Caribbean & British afro-Caribbean	-0.26 (-0.89-0.36)		-0.50 (-1.71-0.70)	
Mixed, other	0.99 (0.34-1.64)		0.99 (0.10-1.88)	
Fathers employment				
Employed	reference group			
Unemployed	1.81 (1.43-2.19)	<0.001	0.97 (0.48-1.45)	<0.001
Housing tenure		<0.001		
Council and private renting, %	reference group			0.128
home owner, %	-1.03 (-1.28- -0.78)		-0.25 (-0.57- -0.07)	
Family income (per £1000 p.a.)	-1.20 (-1.51- -0.88)	<0.001	-0.25 (-0.13-0.62)	0.195
Paternal age	-0.18 (-0.31- -0.04)	0.010		
Predictors (Sweep 3 when child 5 years old)				
*Family context factors*				
Maternal depressive symptoms	0.17 (0.14-0.20)	<0.001	0.08 (0.04-0.11)	<0.001
Marital conflict	1.20 (1.10-1.30)	<0.001	1.09 (0.96-1.22)	<0.001
Child temperament	-0.02 (-0.04- -0.01)	0.008	-0.00 (-0.02-0.02)	0.987
Child gender	-0.12 (-0.28-0.04)	0.140		
*Socioeconomic factors*				
Paternal education		<0.001		0.878
No qualifications	reference group			
School level, Degree or higher	-0.99 (-1.34- -0.65)		0.03 (-0.37-0.43)	
Ethnic background		<0.001		0.001
White	reference group			
Indian, Pakistani, & Bangladeshi	1.01 (0.53-1.49)		1 .09 (0.45-1.72)	
Afro-Caribbean & British afro-Caribbean	-0.59 (-0.95- -0.23)		-0.36 (-1.12-0.40)	
Mixed, other	1.62 (0.76-2.47)		0.73 (-0.13-1.60)	
Fathers employment				
Employed	reference group			
Unemployed	3.01 (2.47-3.56)	<0.001	2.44 (1.66 -3.22)	<0.001
Housing tenure		<0.001		0.366
Council and private renting, %	reference group			
home owner, %	-1.41 ( -1.65- -1.17)		-0.18 (-0.57- 0.21)	
Family income (per £1000 p.a.)	-2.08 (-2.48- -1.69)	<0.001	-0.68 (-1.12 - -0.24)	0.002
Paternal age	-0.11 (-0.25 - 0 .02)	0.088		
Predictors (Sweep 4 when child 7 years old)				
*Family context factors*				
Maternal depressive symptoms	0.18 (0.14-0.22)	<0.001	0.09 (0.04-0.15)	0.001
Marital conflict	1.08 (0.97-1.19)	<0.001	1.00 (0.84-1.15)	<0.001
Child temperament	-0.02 (-0.04- -0.00)	<0.001	-0.00 (-0.02-0.01)	0.713
Child gender	-0.14 (-0.31-0.04)	0.129		
*Socioeconomic factors*				
Paternal education		0.019		
No qualifications	reference group			
School level, Degree or higher	-0.66 (-1.04 - -0.28)			
Ethnic background		0.001		0.589
White	reference group			
Indian, Pakistani, & Bangladeshi	1.20 (0.59-1.81)		0.32 (-0.37-1.01)	
Afro-Caribbean & British afro-Caribbean	-0.01 (-0.68-0.67)		-0.19 (-1.20-0.83)	
Mixed, other	1.35 (0.15-2.55)		0.87 (-0.68-2.43)	
Fathers employment				
Employed	reference group			
Unemployed	2.78 (2.20-3.37)	<0.001	2.40 (1.44-3.37)	<0.001
Housing tenure		<0.001		0.066
Council and private renting, %	reference group			
home owner, %	-1.28 (-1.59- -0.97)		-0.40 (-0.83 - -0.03)	
Family income (per £1000 p.a.)	-2.01 (-2.43- -1.58)	<0.001	-0.61 (-1.10- -0.13)	0.014
Paternal age	-0.10 (-0.26-0.06)	0.229		

^a^ Sample size ranged from 4612 to 12,396 due to missing data in the variables

^b^ Sample size range was 3833 to 6831

#### Multivariable regression analysis

After adjusting for factors that were significantly associated (*p* < 0.01) with paternal depressive symptoms in a multivariable regression (Table 3), maternal depressive symptoms, higher marital conflict and paternal unemployment were significantly associated with higher levels of paternal depressive symptoms at all sweeps. When the cohort children were 9 months old, no qualifications and living in rented housing were significantly associated with higher levels of paternal depressive symptoms. Indian, Pakistani and Bangladeshi ethnicity was associated with higher levels of paternal depressive symptoms compared to white fathers at ages 3 and 5 years. Finally, at 5 and 7 years, lower family income was associated with higher levels of depressive symptoms amongst fathers.

The overall models’ fit R^2^ values were 13, 18, 19 and 16 %.

##### Longitudinal repeated measures model

We fitted a mixed effects model where the outcome was paternal depressive symptoms and included time point, maternal depressive symptoms, marital conflict, paternal education, paternal ethnic background, employment status, family housing and household income as fixed effects. This included a random intercept for each individual and a random slope for time effect to allow for correlation between the repeated measures. Table 4 shows that paternal depressive symptoms decreased over time, with the decrease from sweep 1 to sweep 2 being the steepest. It also shows that maternal depressive symptoms, higher marital conflict and paternal unemployment were highly significantly associated with higher levels of paternal depressive symptoms across all sweeps (*p* < 0.001). Family housing and income were also significantly associated with paternal depressive symptoms across all sweeps.

**Table 4:** Longitudinal repeated measures mixed effects model showing the associations between predictors and paternal depressive symptoms in the Millennium Cohort Study across all time points ^a^

Predictors from all sweeps	Coefficient (95 % CI)^b^	*p*
*MCS wave*		
Wave 1	reference group	
Wave 2	-0.49 (-0.61 - -0.37)	<0.001
Wave 3	-0.44 (-0.56 - -0.31)	<0.001
Wave 4	-0.38 (-0.49 - -0.22)	<0.001
*Family context factors*		
Maternal depressive symptoms	0.08 (0.06 - 0.10)	<0.001
Marital conflict	0.84 (0.77 - 0.91)	<0.001
*Socioeconomic factors*		
Paternal education		0.522
No qualifications	reference group	
School level, Degree or higher	-0.10 (-0.40 - 0.20)	
Ethnic background		
White	reference group	
Indian, Pakistani, & Bangladeshi	0.56 (0.05 - 1.07)	0.031
Afro-Caribbean & British afro-Caribbean	-0.74 (-1.23 - 0.25)	0.003
Mixed, other	0.76 (-0.01 - 1.53)	0.053
Fathers employment		
Employed	reference group	
Unemployed	1.13 (0.70 - 1.56)	<0.001
Housing tenure		0.001
Council and private renting, %	reference group	
home owner, %	-0.41 (-0.66 - -0.17)	
Family income (per £1000 p.a.)	-0.31 (-0.58 - -0.06)	0.022

^a^ Finite population correction factor could not be fitted onto model, but all other required weights (accounting for clustering, attrition/non-response and oversampling for ethnic minorities/disadvantaged participants) were incorporated

^b^ Sample size 4766

## Discussion

Using a nationally representative sample of UK fathers, the current study has found the prevalence of paternal depressive symptoms to be 3.6 % when cohort children were 9 months old, 1.2 % at 3 years old, 1.8 % at 5 years old and 2.0 % at 7 years old. This suggests a decrease in paternal depressive symptoms from the time that their children were 9 months to 3 years old and a very slight increase for the subsequent time points until 7 years old. Maternal depressive symptoms followed the same pattern and reduced from 9 months to 3 years old, and the reduction was greater among mothers compared to fathers. These findings report prevalence at the lower end of the range compared to previous estimates that extend from 1.2 to 25.5 % [9, 12, 14]. Our findings showed slightly higher paternal depressive symptoms compared to the LSAC study of fathers which used the same measure (K6) and cut-off which make our results directly comparable [13], and our finding are extended to children aged up to 7 years old. Taken together, the findings suggest that a small proportion of fathers experience significant levels of depressive symptoms throughout the first 7 years of child’s life, and as with maternal postnatal depression, symptoms were most common in the first year after birth. However, the peak was smaller and the decline was slower than that seen amongst mothers. Thus, it is a possibility that fathers could also suffer from postnatal depression. To our knowledge, this is the first study that has investigated paternal postnatal depressive symptoms in such a large representative cohort with fathers of children aged up to 7 years old.

The association of maternal with paternal depressive symptoms is consistent with previous literature and suggests that fathers are more likely to experience depressive symptoms if their partner also has depression [9, 19]. We found that higher maternal depressive symptoms were associated with higher paternal depressive symptoms across time. Marital conflict was also associated with higher paternal depressive symptoms as predicted and consistent with previous literature [16, 20, 21]. However, child temperament was not significantly associated with paternal depressive symptoms [22, 23], which suggests that within the family context, there are associations with maternal factors but not with child characteristics.

Socio-economic factors such as low income and living in rented accommodation were found to be significantly associated with depressive symptoms, suggesting that fathers in families facing socioeconomic deprivation are more at risk of depressive symptoms, and which replicates the previous literature [26, 43]. Paternal unemployment was strongly and consistently associated with paternal depressive symptoms. This is consistent with previous literature on adult men and findings from one large cohort study of fathers in the United States with children aged 5–17 years old [14, 44]. However, to our knowledge this is the first study to show this finding in a national representative sample in the UK with fathers of children aged between 9 months to 7 years old. These findings suggest that unemployment is a significant risk factor that needs acknowledging by health care providers when it comes to treating depression in fathers of young children, and is particularly salient given the current economic climate.

Fathers with no qualifications reported more depressive symptoms only at 9 months compared to fathers with school level or university level education, which suggests that education was only associated with depressive symptoms during the first year of a child’s life. Similar findings were reported by Bergstrom et al. [26] in a Swedish sample of first time fathers when their infant was 3 months old. The association between ethnicity and paternal depressive symptoms were found when the child was 3 and 5 years old, and suggested that fathers from an Indian, Pakistani, or Bangladeshi background were more likely to have higher depressive symptoms compared to Caucasian fathers. In a study that also used the MCS sample in relation to maternal depression, ethnic density was reported as a major factor associated with maternal depression in mothers from different ethnic backgrounds. Living in an area with higher density was a protective factor for mothers from some backgrounds but the results were not conclusive [27]. In the current sample, the Indian, Pakistani or Bangladeshi ethnic group had a far larger sample size (*n* = 1255) compared to Afro-Caribbean (*n* = 337) and mixed other (356), therefore these results should be interpreted with caution.

The current study has a number of strengths. Firstly, the data consisted of a representative and large sample size of the UK population with a good response rate. The study was unique in that it has data on such a large sample of fathers making them comparable to mothers [45]. The MCS also use well validated and reliable measures [35]. Secondly, sampling weights were used to account for attrition rates that might have affected the results. In addition to this, research indicates that even when drop outs are considered, associations found in regression models are still robust with such large cohort studies [46], suggesting our findings to be robust.

However, the study has a number of limitations. Firstly, depressive symptoms at the first sweep were measured at 9 months. Ideally a measure of depressive symptoms in the more immediate post-natal months following birth could have been more ideal, but we were constrained by available data. Depressive symptoms were also measured differently in the first sweep (Rutter Scale) to the later sweeps (K6), which could have influenced the decrease in depressive symptoms detected from sweep 1 to sweep 2. There was also a slight increase in depressive symptoms from sweeps 2 to 4 where the same scale (K6) was used to measure depressive symptoms. While the use of a different measure at sweep 1 was not ideal, it was important to assess depressive symptoms at this time, and secondary analyses are constrained by the available data. The mean scores and prevalence from the recalibrationed Rutter scale at sweep 1 are similar mean scores of mothers’ and fathers’ depressive symptoms that have been reported by other studies using the K6 questionnaires [47, 48], suggesting that the recalibration of the scale was appropriate. Secondly, as a questionnaire was used to indicate depressive symptoms, reference to clinical diagnoses of depressive episodes could not be made. Although there are limitations with relying on self-report questionnaire measures to investigate depressive symptoms and it is arguably better to administer clinical interviews methods such as the Structural Clinical Interview for DSM-II-R (SCID) [49], conducting such clinical interviews with a large sample size would be costly, time-consuming and would impose burden on participants. Cairney and colleagues compared the K6 questionnaire with clinical interview diagnosis of current depression and reported it to be an “excellent screening instrument, especially for current depression” [50]. This suggests that the use of questionnaires such as the K6 in large epidemiological studies are informative of current depression and to some extent indicates impairments related to a clinical depressive episode. Thirdly, despite significant findings, our coefficient effect sizes of the associations and overall model fit values were small. Although this could be problematic, recruitment of clinically depressed fathers into studies with their children has been reported as particularly challenging [51–53]. Thus, findings from large cohort studies such as the MCS on paternal depressive symptoms offers understanding about the possible risk factors [54, 55]. These findings, if replicated in clinical studies with more in-depth measures, could suggest targets for development of clinical interventions with fathers and mothers.

Finally, the findings are limited to full-time resident fathers only and cannot be applied to general population of fathers who are separated, divorced or non-resident. Research indicates that non-resident fathers have higher levels of depressive symptoms compared to resident fathers who are married or cohabiting [13, 56]. Therefore, the current finding might under-represent the prevalence of depressive rates amongst fathers in general. Although we were interested in investigating this, we were unable to due to the lack of data available on symptoms of depression for part-time and non-resident fathers.

Despite these limitations, the study has considerable strengths, and the findings are timely and add to theoretical understanding of paternal depression. The next-step would be to take associated factors and test for causal relations by using experimental design and longitudinal data analysis to determine if associated factors cause paternal depression or proceed after paternal depression. This could inform better targeted interventions and treatment for fathers with depression. In addition to this, future work should account for other genetic and environmental risk factors associated with paternal depressive symptoms that could not be tested in the current study such as family history of depression, fathers’ past depressive episodes and paternal substance abuse [10].

## Conclusions

In conclusion, there are two key findings from the current study. Firstly, the prevalence of paternal depressive symptoms decreased from the first year (9 months old) to 3 years old, in the same pattern as maternal depressive symptoms. Secondly, paternal depressive symptoms were associated with unemployment, marital conflict and maternal depressive symptoms at every time point, with low socioeconomic variables also playing a role. The key contribution to current theoretical knowledge is that paternal unemployment might be an important factor for paternal depression. Currently in the UK, healthcare cost of depressed fathers is comparable to mothers but interventions are still primarily tailored for mothers [57, 58]. In light of our findings, we would recommend a more family centred approach. Primary health care services, unemployment officers, job centres and health care professionals involved in post-natal care should be aware that fathers of young children are at risk of depression and provide useful services to support such fathers accordingly. Provided suitable, accessible and effective services were available, the systematic screening of new fathers could be implemented alongside the systematic screening of new mothers by health visitors.

## Abbreviations

K6, Kessler 6; LSAC, Longitudinal Study of Australian Children; MCS, Millennium Cohort Study; MEPS, Medical Expenditure Panel Survey; PHQ-2, Patient Health Questionniare-2; UK, United Kingdom
